# The Lifespan Extension Ability of Nicotinic Acid Depends on Whether the Intracellular NAD^+^ Level Is Lower than the Sirtuin-Saturating Concentrations

**DOI:** 10.3390/ijms21010142

**Published:** 2019-12-24

**Authors:** Nae-Cherng Yang, Yu-Hung Cho, Inn Lee

**Affiliations:** 1 Department of Nutrition, Chung Shan Medical University, Taichung 402, Taiwan; 2 Department of Nutrition, Chung Shan Medical University Hospital, Taichung 402, Taiwan

**Keywords:** nicotinic acid, calorie restriction mimetic, NAD^+^, lifespan, *C. elegans*, Hs68 cells

## Abstract

Calorie restriction can extend lifespan by increasing intracellular nicotinamide adenine dinucleotide (NAD^+^), thereby upregulating the activity of sirtuins (*Caenorhabditis elegans* Sir-2.1; human SIRT1). Nicotinic acid (NA) can be metabolized to NAD^+^; however, the calorie restriction mimetic (CRM) potential of NA is unclear. This study explored the ability and mechanism of NA to extend the lifespan of human Hs68 cells and *C. elegans*. We found that NA can efficiently increase the intracellular NAD^+^ levels in Hs68 cells and *C. elegans*; however, NA was only able to extend the lifespan of *C. elegans*. The steady-state NAD^+^ level in *C. elegans* was approximately 55 μM. When intracellular NAD^+^ was increased by a mutation of *pme-1* (poly (ADP-ribose) metabolism enzyme 1) or by pretreatment with NAD^+^ in the medium, the lifespan extension ability of NA disappeared. Additionally, the saturating concentration of NAD^+^ required by SIRT1 was approximately 200 μM; however, the steady-state concentration of NAD^+^ in Hs68 cells reached up to 460 μM. These results demonstrate that the lifespan extension ability of NA depends on whether the intracellular level of NAD^+^ is lower than the sirtuin-saturating concentration in Hs68 cells and in *C. elegans*. Thus, the CRM potential of NA should be limited to individuals with lower intracellular NAD^+^.

## 1. Introduction

Calorie restriction mimetic (CRM) is a compound that mimics the metabolic, hormonal, and physiological effects of calorie restriction (CR) to produce CR-like effects on longevity and reduce age-related disease [1]. Nicotinamide adenine dinucleotide (NAD^+^) is a coenzyme involved in cellular energy metabolism, adaptive responses, neurodegenerative disorders, and aging [2,3,4]. One of the mechanisms of CR-mediated longevity of organisms is the NAD^+^–sirtuin pathway, which includes an increase of the intracellular NAD^+^ level by CR with subsequent activation of sirtuins (e.g., Sir-2.1 in nematodes and SIRT1 in humans) to extend the lifespan of the organisms [5]. Vitamin B_3_ (also known as niacin) can be metabolized to produce NAD^+^ and may activate sirtuins to promote longevity of organisms. However, the CRM potential of vitamin B_3_ remains unclear.

It is known that nicotinic acid (NA) and nicotinamide (NAM) are two major forms of vitamin B_3_. Studies have found that NA has a good blood lipid-lowering effect and can reduce the progression of atherosclerosis and reduce the risk of cardiovascular events [6], while NAM has been reported to prevent the onset of type 1 diabetes. However, human clinical trials have failed to demonstrate these effects [7]. Our previous study has shown that using FK866, an Nampt (nicotinamide phosphoribosyltransferase; a rate-limiting enzyme in the NAD^+^ synthetic salvage pathway from NAM to NAD^+^) inhibitor, to mimic vitamin B_3_ deficiency in human Hs68 cells can reduce the lifespan of Hs68 cells, suggesting that vitamin B_3_ deficiency can potentially induce aging [8]. On the other hand, it is reasonable to suggest that supplementation with vitamin B_3_ may potentially act as a CRM to extend the lifespan of organisms. Indeed, potential use of vitamin B_3_ as a CRM has been extensively discussed in the literature [9,10]. A major feature of CRM is induction of longevity; however, a previous study has shown that NAM cannot extend the lifespan of C57BL/6J mice [11]. Since NAM itself is an inhibitor of sirtuins [12] and can inhibit poly(ADP-ribose) polymerases (PARP) to induce apoptosis [13], the mechanism of action of NAM may be complex. Indeed, NAM actually increased the lifespan of *Caenorhabditis elegans*, and this effect is obvious even in the absence of sirtuins [14]. Therefore, this study selected NA as the vitamin B_3_ representative molecule to study the CRM potential.

A previous study has shown that NA can extend the lifespan of *C. elegans* through Sir-2.1 [14], suggesting the CRM potential of NA. However, a previous study reported that NA does not to extend the lifespan of BJ cells [15]. In our previous paper, we found that calorie restriction can extend the replicative lifespan of Hs68 cells [5], suggesting that NA may be able to extend the lifespan of Hs68 cells if it has CRM potential. Thus, this study intended to explore the ability and mechanism of NA to extend the lifespan of Hs68 cells and *C. elegans*. We hypothesized that NA can extend the lifespan of Hs68 cells and *C. elegans* via the NAD^+^–sirtuin signaling mechanism. The growth curve of the cumulative population doubling level (CPD) was used to monitor the replicative lifespan of Hs68 cells; the senescence-associated β-galactosidase activity was detected in parallel as a cell senescence marker [16]. The effects of NA on the lifespan of *C. elegans* were assessed according to the lifespan assay and three physiological indexes—pharyngeal pumping, autofluorescence, and body bends, which were used as additional aging markers. The intracellular NAD^+^ levels of Hs68 cells and *C. elegans* were determined by acid extraction, followed by the enzymatic cycling method. Two mutants were used: *sir-2.1* or *pme-1*. Pretreatment with NAD^+^ in the nematode growth medium (NGM) plate was used to reveal the role of Sir-2.1 and the effect of the steady-state level of intracellular NAD^+^ on the ability of NA to extend the lifespan of *C. elegans*. Additionally, a commercially available SIRT1 activity kit was used to determine the saturating concentration of NAD^+^ as a cosubstrate of SIRT1.

## 2. Results

### 2.1. Effects of NA on Intracellular NAD^+^ in Hs68 Cells and C. elegans

**Figure 1:**
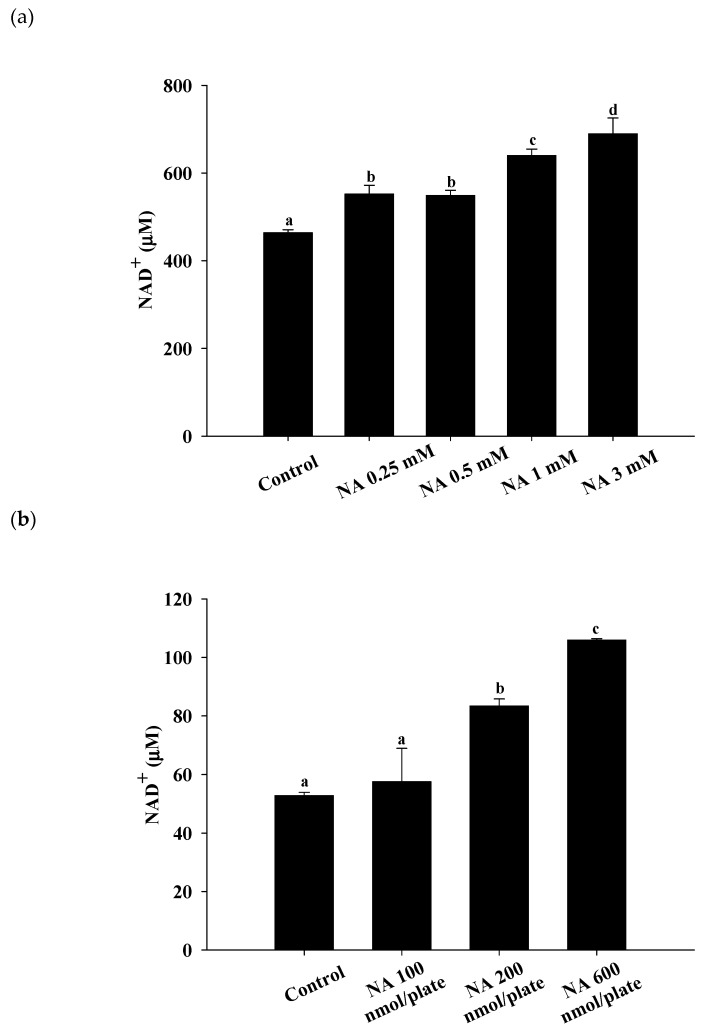
Effects of nicotinic acid (NA) on the intracellular nicotinamide adenine dinucleotide (NAD^+^) levels in Hs68 cells and *Caenorhabditis elegans*. (**a**) Hs68 cells were cultured in medium containing 0, 0.25, 0.5, 1, and 3 mM NA for 24 h. (**b**) *C. elegans* were grown on nematode growth medium (NGM) plates containing 0, 100, 200, and 600 nmol NA/plate in the center of the top surface of NGM for seven days. The intracellular NAD^+^ levels in Hs68 cells and *C. elegans* were measured. Values (mean ± SD, *n* = 3 independent experiments) not sharing a common letter are significantly different (*p* < 0.05).

After Hs68 cells were incubated with various concentrations of NA (0, 0.25, 0.5, 1, and 3 mM) for one day and nematodes were incubated with various dosages of NA (0, 100, 200, and 600 nmol/plate) for seven days, the intracellular NAD^+^ levels were determined by the enzymatic cycling method. It has been reported that adding 1 mM NA in NGM can extend the lifespan of *C. elegans* [14]. However, during the treatment with NA in NGM, only NA molecules on the surface of NGM can be taken up by *C. elegans*. In this study, we directly spiked NA in the LB medium and spread NA onto the center of the top surface of NGM with OP50 bacteria. According the concentration used in a previous report [14], we used 0.5, 1, and 3 mM NA in LB medium and spread 200 μL of the corresponding solutions onto the surface NGM with OP50; thus, the dosages of NA used for the *C. elegans* treatment were 100, 200, and 600 nmol/plate (note: The LB medium would dry out after spreading onto the surface of NGM, thus the dosage unit of nmol/plate was used rather than the molar concentration of NA). The results showed that NA can significantly increase the intracellular NAD^+^ levels in Hs68 cells and *C. elegans*. As shown, NA significantly increased (*p* < 0.05) intracellular NAD^+^ at concentrations from 0.25 to 3 mM in Hs68 cells (Figure 1a) and dosages of 200 and 600 nmol/plate for nematodes (Figure 1b).

### 2.2. Effects of NA on the Replicative Lifespan and Senescence-Associated β-Galactosidase (SA-βG) Activity of Hs68 Cells

**Figure 2:**
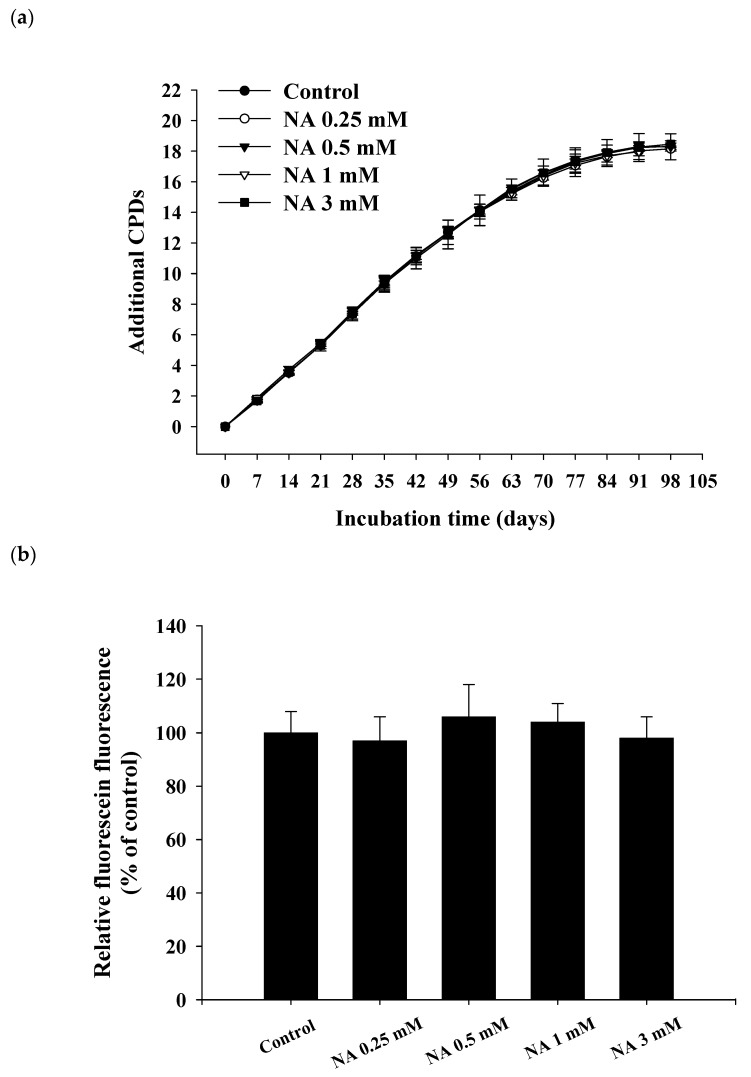
Effects of nicotinic acid (NA) on the replicative lifespan and senescence-associated β-galactosidase (SA-βG) activity. Hs68 cells were serially cultured in medium containing 0, 0.25, 0.5, 1, and 3 mM NA. (**a**) Cumulative growth curves were obtained to determine the replicative lifespan. (**b**) On day 91 of the serial culture, SA-βG activities were measured by the double-substrate method, and the fluorescein fluorescence is shown as a % of the control. Values are mean ± SD, *n* = 3 plates.

To estimate the lifespan of Hs68 cells, cells were incubated with various concentrations of NA (0, 0.25, 0.5, 1, and 3 mM) and the cumulative growth curves were obtained (Figure 2a). During cumulative growth at day 91, the SA-βG activity in Hs68 cells was determined by the double-substrate method [8], i.e., qualitatively by X-Gal staining (Figure S1; see the Supplementary Materials) and quantitatively by relative fluorescein fluorescence (Figure 2b). The results showed that the replicative lifespan or the SA-βG activity was not significantly (*p* > 0.05) influenced by NA, illustrating that NA cannot extend the lifespan of Hs68 cells.

### 2.3. Effects of NA on the Lifespan and Physiological Indexes of C. elegans

**Figure 3:**
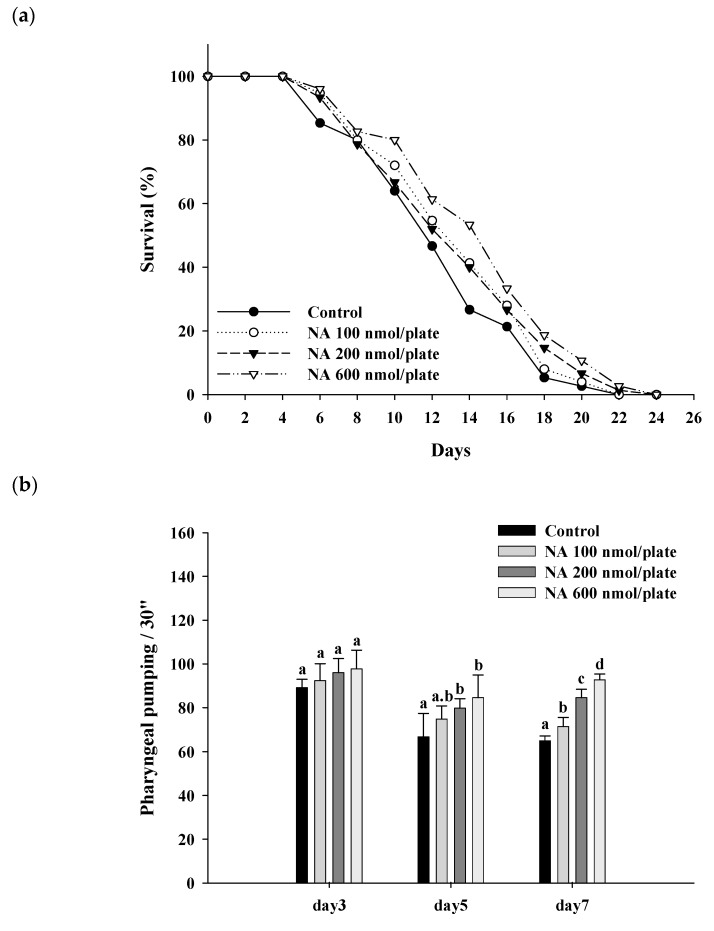

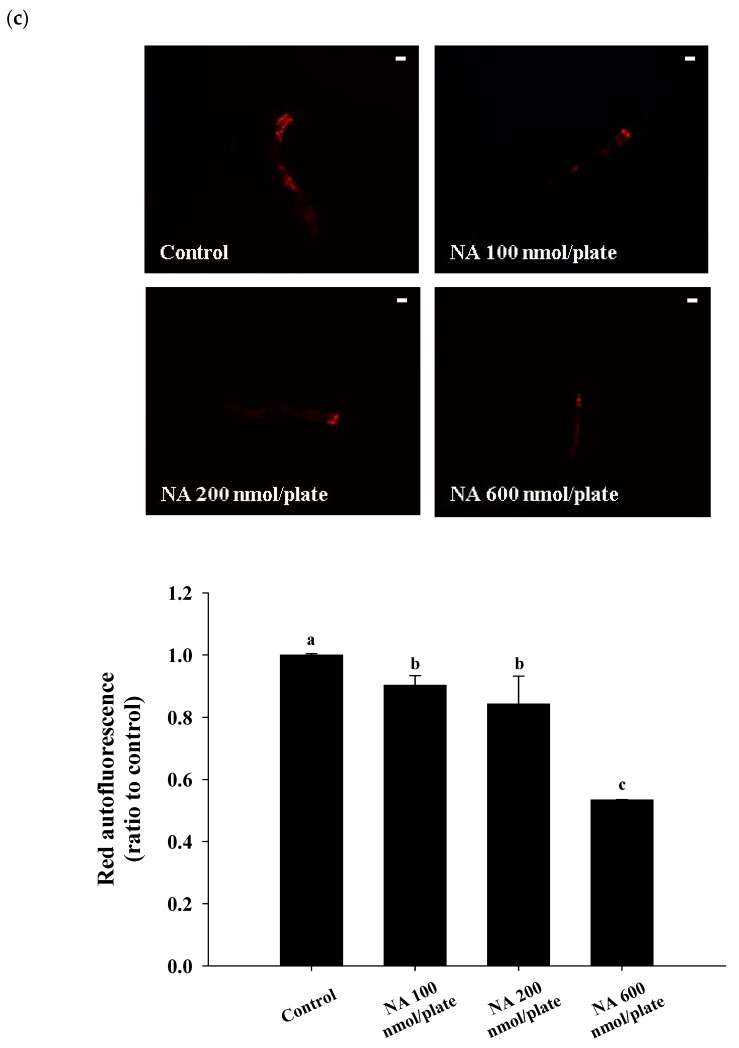

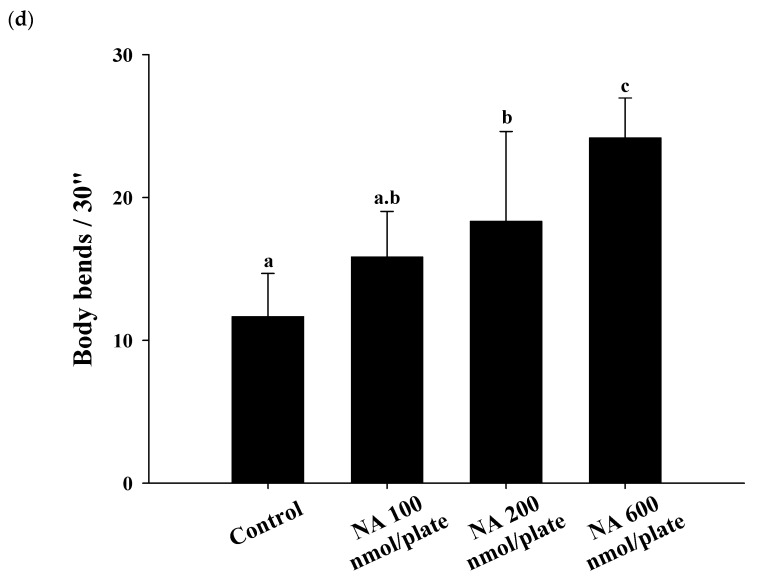
Effects of nicotinic acid (NA) on the lifespan of *C. elegans* and accessory aging markers, including pharyngeal pumping, autofluorescence, and body bends. Nematodes were grown on plates containing various dosages of NA as described in Section 4. (**a**) 90 worms (*n* = 90) for each group were used, counting the number of surviving worms every two days to obtain the lifespan data. (**b**) On the third, fifth, and seventh days of life, the pharyngeal pumping of nematodes (*n* = 10 worms) was determined. (**c**) On the 10th day of life, the red autofluorescence of the nematodes (*n* = 3 worms) was determined; representative images and the corresponding quantification results are shown. (**d**) On the 11th day of life, body bend of the nematodes (*n* = 10 worms) was determined. Values (mean ± SD) not sharing a common letter are significantly different (*p* < 0.05). Scale bar is 100 μm.

Different dosages of NA (0, 100, 200, and 600 nmol/plate) were spread onto the central surface of NGM to test the effects of NA on the lifespan of nematodes. The results showed that NA at a dosage of 600 nmol/plate could significantly (*p* < 0.05) extend the lifespan of nematodes; the average lifespan was increased by 17%, and the median lifespan was increased by four days (Figure 3a; Table S1). Additionally, NA could significantly (*p* < 0.05) increase the pharyngeal pumping of *C. elegans* (Figure 3b), reduce the autofluorescence of *C. elegans* (Figure 3c) and significantly increase the body bends of *C. elegans* in the swimming test (Figure 3d).

### 2.4. Effects of NA on the NAD^+^ Level, Lifespan, and Physiological Indexes of the sir-2.1 Mutant of C. elegans

**Figure 4:**
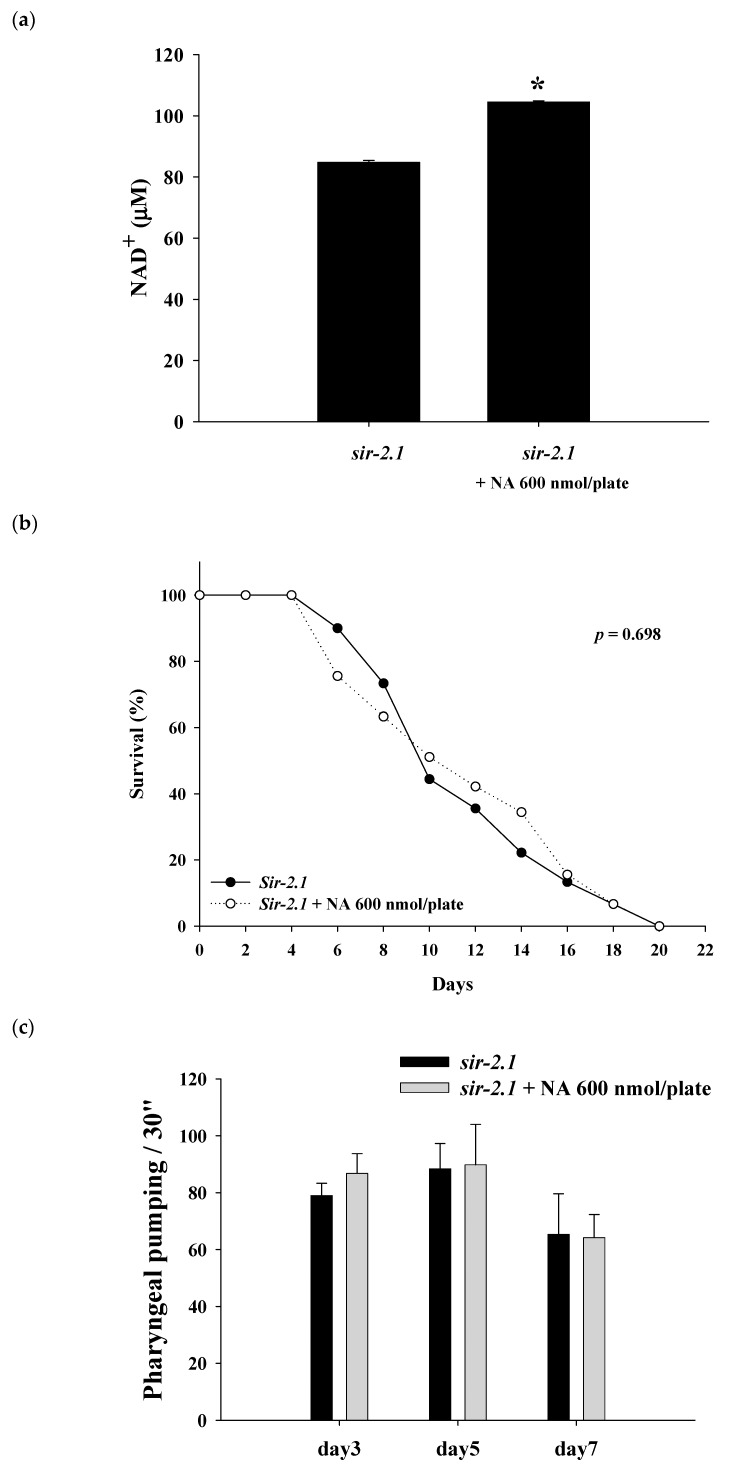

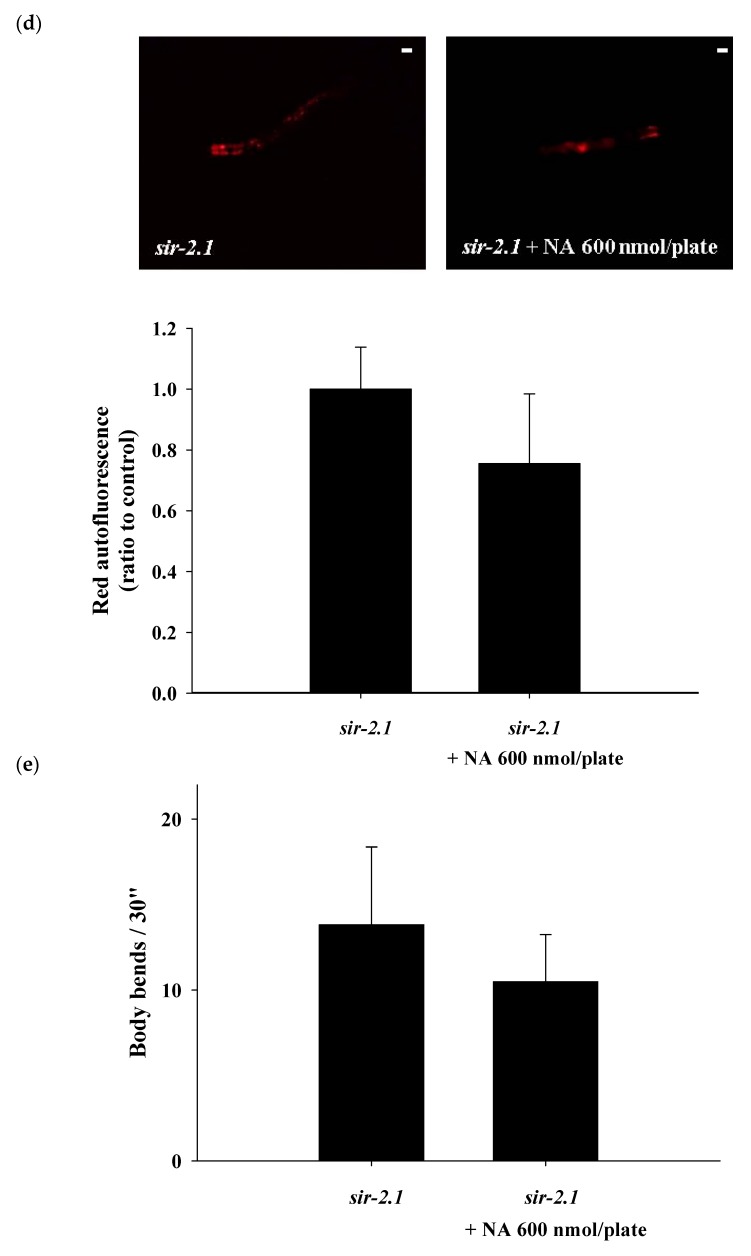
Effects of nicotinic acid (NA) on intracellular NAD^+^, lifespan, pharyngeal pumping, autofluorescence, and body bends of the *sir-2.1* mutants. The mutants were grown on plates with or without 600 nmol/plate NA. (**a**) The intracellular NAD^+^ levels (*n* = 3 independent experiments) and (**b**) lifespan (*n* = 90 worms). (**c**) On the third, fifth, and seventh days of life, pharyngeal pumping (*n* = 10 worms) was performed. (**d**) On the 10th day of life, red autofluorescence (*n* = 3 worms) was measured. (**e**) On the 11th day of life, body bend (*n* = 10 worms) was determined. Values are expressed as the mean ± SD; asterisks * indicate significant changes (*p* < 0.05) compared with the control mutants. Scale bar is 100 μm.

The *sir-2.1* mutants were grown on the plates with or without 600 nmol NA/plate on the surface. After growth for seven days, the nematode homogenates were collected and used to determine the NAD^+^ level. The results showed that the NAD^+^ level was significantly increased in the nematodes treated with NA (Figure 4a). However, NA did not significantly (*p* > 0.05) extend the lifespan of *sir-2.1* mutants (Figure 4b; Table S2). This result showed that the lifespan extension ability of NA disappeared in *sir-2.1* mutants, demonstrating that NA prolongs the lifespan of *C. elegans* via the Sir-2.1 signaling pathway. Moreover, the increase in pharyngeal pumping and body bend and the reduction in autofluorescence induced by NA also disappeared in *sir-2.1* mutants (Figure 4c–e), suggesting that the effects of NA on these aging accessory markers are also mediated by Sir-2.1.

### 2.5. Saturating Concentration of NAD^+^ as a Cosubstrate for SIRT1

**Figure 5:**
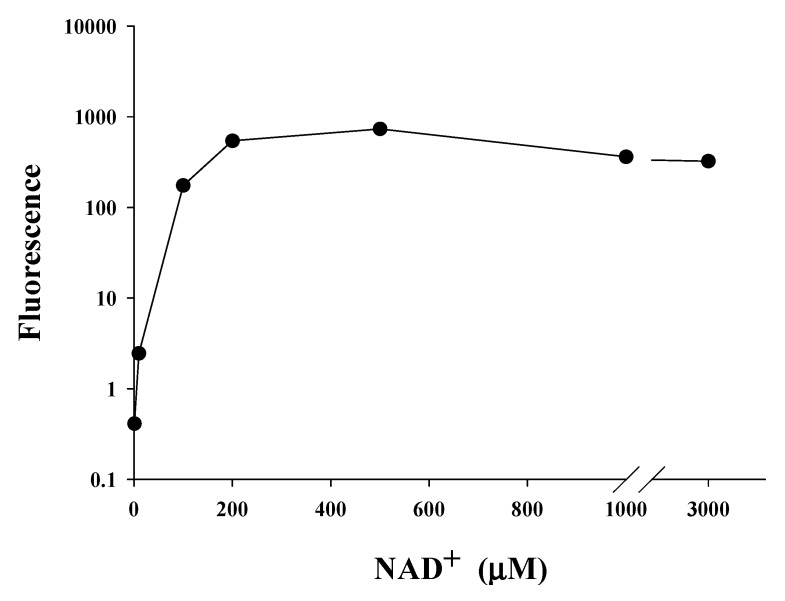
SIRT1-saturating NAD^+^ concentration in vitro. SIRT1 activities were determined in reactions containing various concentrations of NAD^+^ (1, 10, 200, 500, 1000, and 3000 μM NAD^+^, *n* = 3 independent experiments) using a commercialized SIRT1 Fluorescence Resonance Energy Transfer (FRET)-based screening assay kit.

We found that NA could significantly increase the intracellular level of NAD^+^ in *C. elegans* and Hs68 cells; however, NA could only extend the lifespan of *C. elegans*. As shown in the Figure 1a,b, the steady-state levels of intracellular NAD^+^ in Hs68 cells and *C. elegans* were approximately 460 and 55 μM, respectively, which are quite different. We thus hypothesized that the intracellular NAD^+^ level in Hs68 cells is higher than the maximal concentration of NAD^+^ needed for SIRT1; thus, NA cannot increase the SIRT1 activity via an increase of the intracellular NAD^+^ level. The saturating concentration of NAD^+^ for SIRT1 was then determined by the SIRT1 Fluorescence Resonance Energy Transfer (FRET)-based screening assay kit (Cayman, Ann Arbor, MI, USA). The results showed that the enzyme activity reached the maximum level when the NAD^+^ concentration was increased to approximately 200 μM (Figure 5).

### 2.6. Effects of NA on the NAD^+^ Level, Lifespan, and Physiological Indexes of pme-1 Mutants of C. elegans

**Figure 6:**
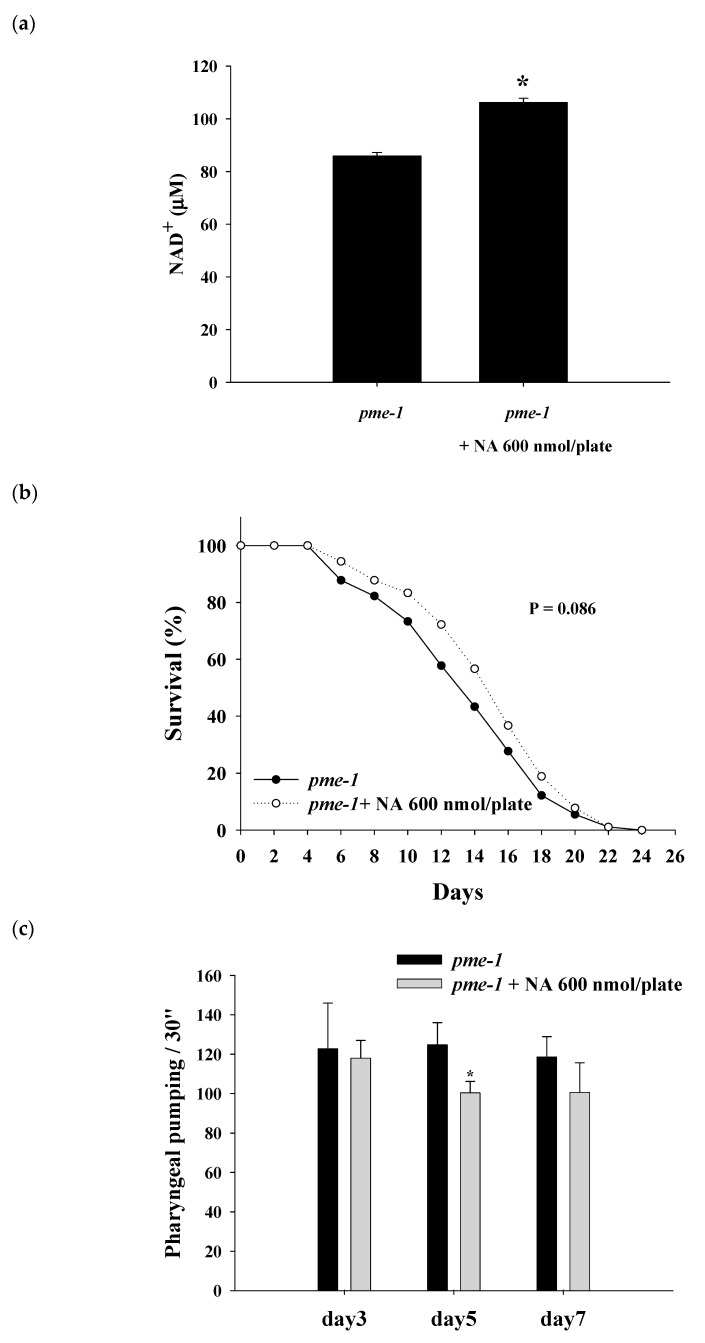

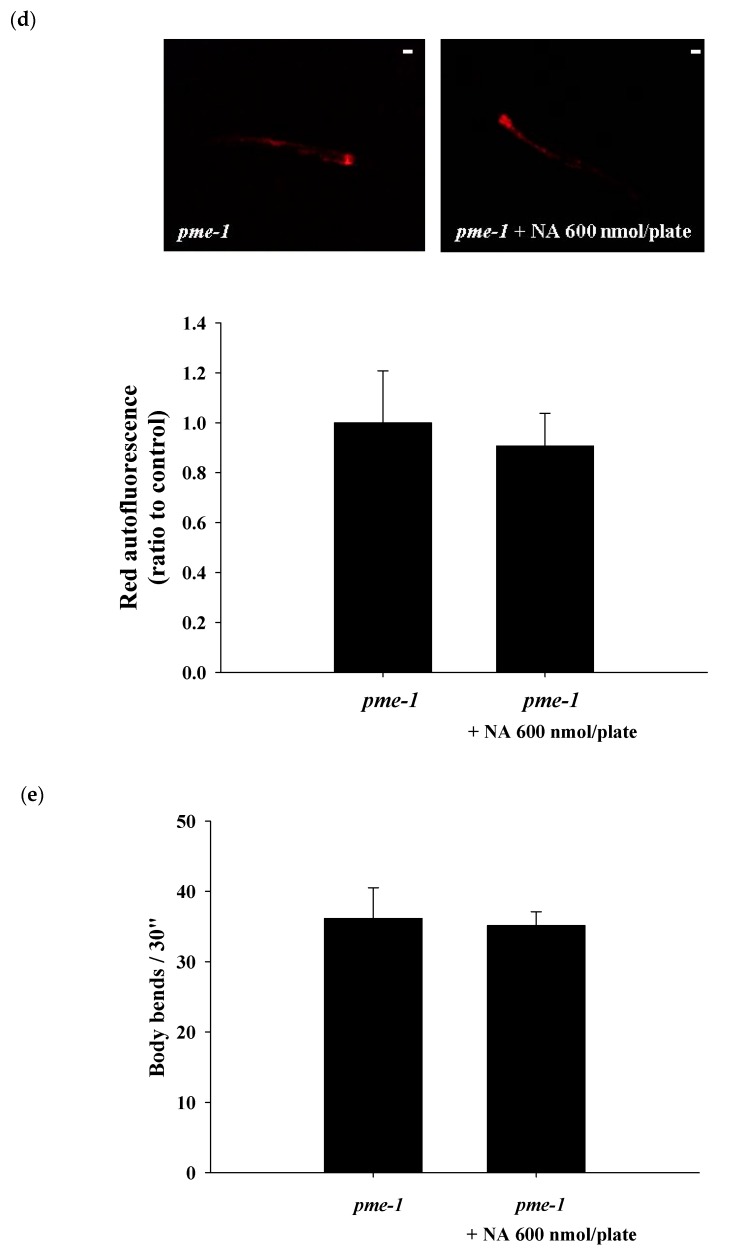
Effects of nicotinic acid (NA) on the level of intracellular NAD^+^, lifespan, pharyngeal pumping, autofluorescence, and body bends of *pme-1* mutants. The mutants were grown on plates with or without 600 nmol NA/plate. (**a**) The intracellular NAD^+^ levels (*n* = 3 independent experiments) and (**b**) lifespan (*n* = 90 worms). (**c**) On the third, fifth, and seventh days of life, pharyngeal pumping (*n* = 10 worms) was performed. (**d**) On the 10th day of life, red autofluorescence (*n* = 3 worms) was measured. (**e**) On the 11th day of life, body bend (*n* = 10 worms) was determined. Values are expressed as the mean ± SD; asterisks * indicate significant changes (*p* < 0.05) compared with the control mutants. Scale bar is 100 μm.

The results showed that the NAD^+^ level was significantly increased in *pme-1* mutants after treatment with NA (Figure 6a). The steady NAD^+^ level in the *pme-1* mutants was 86 ± 1.3 μM, which was approximately 156% higher than the 55 μM level of wild-type *C. elegans*, thus confirming an increase in intracellular NAD^+^ in the *pme-1* mutants. However, NA did not significantly (*p* = 0.068) extend the lifespan of the *pme-1* mutants (Figure 6b; Table S3), suggesting that the lifespan extension ability of NA was associated with the intracellular NAD^+^ level. Moreover, the increase in pharyngeal pumping and body bend and the reduction in autofluorescence induced by NA essentially disappeared in *pme-1* mutants (Figure 6c–e).

### 2.7. Effects of NA on the NAD^+^ Level, Lifespan, and Physiological Indexes in Normal C. elegans with an Increased Intracellular NAD^+^ Level

**Figure 7:**
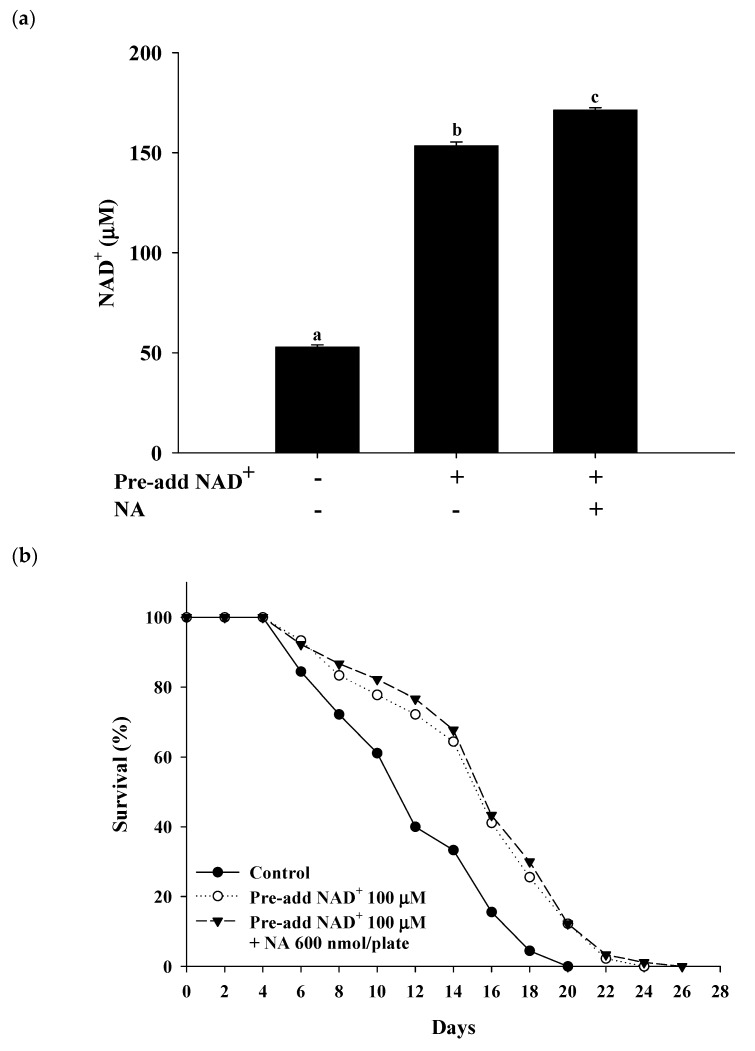

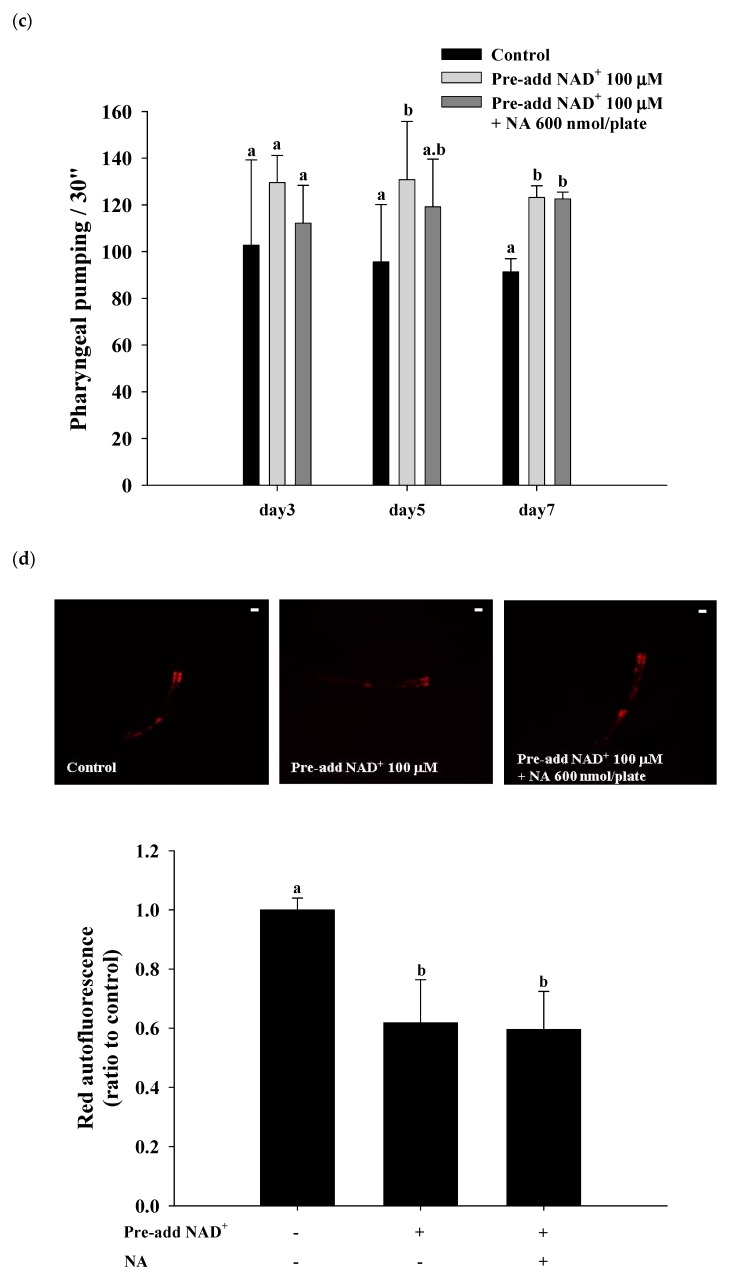

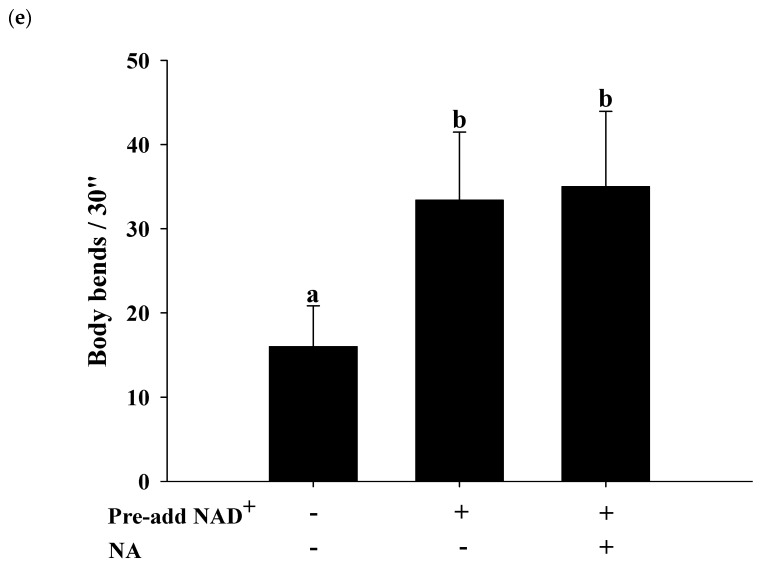
Effects of nicotinic acid (NA) on the level of intracellular NAD^+^, lifespan, pharyngeal pumping, autofluorescence, and body bends in wild-type *C. elegans* grown on the NGM plates pretreated with NAD^+^. *C. elegans* were grown on the plates pretreated with 100 μM NAD^+^ in NGM and on plates with or without 600 nmol NA/plate on NGM. (**a**) The intracellular NAD^+^ levels (*n* = 3 independent experiments) and (**b**) lifespan (*n* = 90 worms). (**c**) On the third, fifth, and seventh days of life, pharyngeal pumping (*n* = 10 worms) was performed. (**d**) On the 10th day of life, red autofluorescence (*n* = 3 worms) was measured. (**e**) On the 11th day of life, body bend (*n* = 10 worms) was determined. Values (mean ± SD) not sharing a common letter are significantly different (*p* < 0.05). Scale bar is 100 μm.

The effects of NA on wild-type *C. elegans* with an increased NAD^+^ level were tested by pretreatment with NAD^+^ in the NGM plates. Hashimoto et al. [17] reported that the addition of 10, 100, and 1000 μM NAD^+^ to NGM can extend the nematodes’ lifespan. The authors found that 100 and 1000 μM NAD^+^ in NGM had a similar lifespan extension effect in nematodes, suggesting that adding 100 μM NAD^+^ in NGM can achieve the maximal concentration of the intracellular NAD^+^ required for Sir-2.1. In this study, we used the same method of pretreatment with 100 μM NAD^+^ in NGM and found that the intracellular NAD^+^ levels in *C. elegans* were increased by approximately 290% over those in the control to approximately 153 μM at a dosage of 600 nmol/plate of NA. Under these conditions, NA still significantly increased NAD^+^ in nematodes (Figure 7a); however, the lifespan extension ability of NA in *C. elegans* disappeared (Figure 7b; Table S4). Moreover, the increase in pharyngeal pumping and body bends and the reduction in autofluorescence induced by NA disappeared (Figure 7c–e). These results demonstrate that the intracellular NAD^+^ levels of the nematodes can influence the lifespan extension ability of NA.

## 3. Discussion

To evaluate the CRM potential of nicotinic acid (NA), this study aimed to explore the ability and mechanism of NA to extend the lifespan of Hs68 cells and *C. elegans*. The results showed that NA cannot extend the lifespan of Hs68 cells; however, NA did significantly extend the lifespan of nematodes. NA ameliorated pharyngeal pumping, body bend, and autofluorescence, thus confirming the health benefit and antiaging properties of NA in *C. elegans*. By contrast, the effects of NA on the lifespan extension and physiological improvements disappeared when the intracellular NAD^+^ levels were increased by two methods: (1) the use of the *pme-1* mutants and (2) the direct addition of NAD^+^ to NGM plates. These results demonstrated that the lifespan-extending ability of NA was tightly associated with the intracellular level of NAD^+^ in *C. elegans*. Importantly, we found that the saturating concentration of NAD^+^ for SIRT1 was approximately 200 μM; however, the steady-state concentration of NAD^+^ in Hs68 cells reached up to 460 μM, which was substantially higher than the maximal NAD^+^ concentration required for SIRT1. These results demonstrated that the lifespan extension ability of NA depends on whether the intracellular NAD^+^ level was lower than the sirtuin-saturating concentration.

A previous study indicated that the Michaelis constant (Km) of NAD^+^ for SIRT1 was approximately 390 μM [18] (note that the saturating concentration = 2 × Km); however, other studies reported that the Km or the saturating concentration of NAD^+^ for SIRT1 were within a similar range detected in the present study. For examples, Km has been reported to be approximately 95 [19,20] or 125 μM [21]; the saturating concentration has been reported to be approximately 200–250 μM [22,23,24]. Our results confirmed that the saturating concentration of NAD^+^ required for SIRT1 was approximately 200–250 μM. On the other hand, we found that the steady-state level of NAD^+^ in *C. elegans* was approximately 55 μM. To the best of our knowledge, there is no data in the literature on the Km value or the saturating concentration of NAD^+^ for Sir-2.1 of *C. elegans*. There is no commercially available Sir-2.1 protein on the market; hence, the saturating concentration of NAD^+^ for Sir-2.1 was not determined in this study. However, when the intracellular NAD^+^ level was increased to approximately 153 μM by adding 100 μM NAD^+^ to the NGM plates, the lifespan of *C. elegans* was not extended by NA, suggesting that the saturating concentration of NAD^+^ for Sir-2.1 may be approximately 150 μM. A previous study has shown that the steady-state concentration of NAD^+^ in the *pme-1* mutant strains is approximately 1.5 times higher than that in wild-type *C. elegans* [25]; our results are consistent with these data. Moreover, the lifespan extension ability of NA had a marginal efficacy (i.e., *p* = 0.086) when the intracellular NAD^+^ level reached up to 86 μM in the *pme-1* mutants, suggesting that the saturating concentration of NAD^+^ for Sir-2.1 may be higher than 86 μM. These results support our hypothesis that the intracellular NAD^+^ level is lower than the saturating concentration of NAD^+^ for Sir-2.1, and thus, NA can extend the lifespan of *C. elegans*. By contrast, the steady-state NAD^+^ level was substantially higher than the saturating concentration of NAD^+^ for SIRT1 in Hs68 cells, and thus, NA cannot extend the lifespan of Hs68 cells via the NAD^+^–SIRT1 signaling mechanism. Therefore, this study successfully answered the question of why NA can extend the lifespan of nematodes but cannot extend the lifespan of Hs68 cells.

We have previously demonstrated that FK866 can induce a vitamin B_3_ deficiency, mimicking the situation in Hs68 cells [8]. We found that the intracellular NAD^+^ level can be significantly decreased in parallel to a significantly shortened lifespan in Hs68 cells treated with FK866. Interestingly, the FK866-induced lifespan shortening of Hs68 cells can be antagonized by cotreatment with NA. An FK866-induced decrease in SIRT1 activity in Hs68 cells was also antagonized by cotreatment with NA [8]. The results suggested that NA can extend the lifespan of Hs68 cells and activate SIRT1 when the intracellular NAD^+^ level is considerably reduced in Hs68 cells by treatment with FK866. These previous results support the mechanism proposed in the current study.

Furthermore, numerous studies have demonstrated that human cells under conventional culture conditions have NAD^+^ levels generally higher than 200–250 μM [26,27,28,29,30]; for example, Hara et al. [26] reported that the steady-state levels of NAD^+^ are 503 ± 104 μM in HEK293 cells, 546 ± 46 μM in HeLa cells, and 597 ± 90 μM in HL60 cells. However, regular cell culture medium contains sufficient vitamin B_3_ to produce NAD^+^. For example, the medium for Hs68 cells, i.e., Dulbecco’s modified Eagle medium (DMEM), contains 4 mg/L NAM. DMEM also contains 16 mg/L tryptophan, which may increase the amount of NAD^+^ via the de novo biosynthesis pathway [4]. Therefore, it is reasonable to assume that cultured cells may have higher levels of intracellular NAD^+^ than in vivo. This led to a question whether intracellular NAD^+^ in the human body is lower than the saturating concentration of NAD^+^ for SIRT1. It has been previously reported that under normal conditions, the concentration of NAD^+^ in human lymphocytes is 401 ± 128 μM [31] and the concentration of NAD^+^ in the human brain tissue is 300 ± 20 μM [32]. These results suggest that the intracellular NAD^+^ level is still substantially higher than the SIRT1-saturating concentrations. These results suggest that the potential of NA as a CRM in the human body may be realized only in individuals with lower intracellular NAD^+^, e.g., vitamin B_3_-deficient individuals or the elderly.

However, it should be mentioned that the health benefits of vitamin B_3_ may be mediated by different the mechanisms rather than the NAD^+^–SIRT1 pathway. For example, it is known that there are seven different sirtuins (i.e., SIRT1-7) in mammals [4]. The Km values for NAD^+^ vary widely between sirtuins; for example, the Km for SIRT2 is 83 μM [33], for SIRT3 is 880 μM [34], for SIRT4 is 35 μM [35], for SIRT5 is 980 μM [36], and for SIRT6 is 26 μM [37]. Thus, there still are certain sirtuins with a saturating concentration obviously higher than 200–250 μM. Moreover, it is possible that an increase in intracellular NAD^+^ by vitamin B_3_ may have certain health benefits via other mechanisms in addition to the effects on sirtuins. For example, a recent paper reported that NAM cannot prolong the lifespan of C57BL/6J mice; however, NAM still had certain health benefits via the hepatic glucose metabolism regulation and oxidative stress reduction [11]. The NAD^+^ signaling mechanisms relating to the therapeutic potential of NAD^+^-boosting molecules including NA, NAM, nicotinamide riboside (NR; a new type of vitamin B_3_ molecule), and nicotinamide mononucleotide (NMN; an NAD^+^ precursor) have been reviewed elsewhere [38].

## 4. Materials and Methods

### 4.1. Materials

All chemicals used were of analytical grade. NaCl, KCl, NaOH, MgCl_2_, Na_2_HPO_4_, KH_2_PO_4_, NaHCO_3_, Na_2_CO_3_, HCl, dimethylsulfoxide (DMSO), and formaldehyde were purchased from Merck (Darmstadt, Germany). NA, NAD^+^, potassium ferricyanide, potassium ferrocyanide, fluorescein, glutaraldehyde, 3-(4,5-dimethylthiazol-2-yl)-2,5-diphenyltetrazolium (MTT), alcohol dehydrogenase (ADH), N-ethyldibenzopyrazine ethyl sulfate (commonly referred to as phenazine ethosulfate), 5-bromo-4-chloro-3-indolyl-β-D-galactopyranoside (X-Gal), ampicillin sodium salt, fluorodeoxyuridine (FUdR), and cholesterol were obtained from Sigma (St. Louis, MO, USA). Ethanol, glycerol, MgSO_4_, and CaCl_2_ were purchased from J.T. Baker^®^ (Phillipsburg, NJ, USA). Fluorescein di(β-D-galactopyranoside) (FDG) was purchased from Molecular Probes (Eugene, OR, USA). American bacteriological agar and yeast extract was obtained from Conda (Madrid, Spain). Dulbecco’s modified Eagle medium (DMEM), fetal bovine serum (FBS), nonessential amino acids (NEAA), penicillin–streptomycin, and sodium pyruvate were obtained from Hyclone^TM^ (Boston, MA, USA). Vegetable peptone and tryptone were obtained from Fluka (Buchs, Switzerland). Human fibroblast Hs68 cells (ATCC^®^ CRL-1635™) were purchased from the Cell Culture Center of the Food Industry Research and Development Institute (Hsinchu, Taiwan). The wild-type *C. elegans* strain N2 was a gift from the *C. elegans* core facility (National Tsing Hua University, Taiwan). The VC199 *sir-2.1(ok343)VI* and RB1042 *pme-1(ok988)* mutants were obtained from the *Caenorhabditis* Genetics Center (University of Minnesota, MN, USA).

### 4.2. Cell Culture

Hs68 cells were routinely cultured in Dulbecco’s modified Eagle medium (DMEM, Gibco, Grand Island, NY, USA) in 75 cm^2^ flasks with 25 mM (4.5 mg/mL) glucose, 10% fetal bovine serum, and 1.0 mM pyruvate at 37 °C in a humidified incubator under 5% CO_2_. Hs68 cells is one of a series of the human foreskin fibroblast lines developed at the Naval Biosciences Laboratory (NBL) in Oakland, CA. Hs68 cells were obtained from an apparently normal Caucasian newborn male with a finite lifespan but may have a low level of aspartoacylase activity.

### 4.3. Determination of the Intracellular NAD^+^ Level in Hs68 Cells

Intracellular NAD^+^ in Hs68 cells was determined by the acid extraction method with subsequent enzymatic cycling [8]. Briefly, the cells were cultured in a 10 cm dish at 1.5 × 10^5^ cells per dish. After 24 h of cell attachment, the cells were incubated with NA for 24 h. Cells were trypsinized and collected at approximately 5 × 10^5^/vial (pooled three dishes of cells). The cell pellet was extracted by adding 500 μL ice-cold 7.0% perchloric acid (PCA). A portion of the extract (i.e., 100 μL) was neutralized with an equal volume of a 1 M K_2_HPO_4_ solution. The level of NAD^+^ was determined using a 96-well microplate in 100 μL reaction mixture per well (*n* = 3). The reaction mixture contained 10 μmol Tris-HCl (pH 8.0), 0.4 μmol PES, 0.05 μmol MTT, 0.03 mg ADH, 60 μmol ethanol, and 20 μL cell extract. The rate of increase in absorbance at 570 nm within 8 min was detected by a microplate reader (Micro Qunat, BioTek, Winooski, VT, USA) and calibrated using commercial NAD^+^ dissolved in the neutralized acid solution in the micromolar range used as the standard. The intracellular NAD^+^ level (μM) = the measured NAD^+^ (μM) × the dilution factor during neutralization (i.e., twofold) × 500 μL/2.55 μL, where 500 μL is the volume of PCA solution and 2.55 μL is the cell volume of 5 × 10^5^ cells (note: 1 × 10^6^ of Hs68 cells is approximately 5.1 μL [5]).

### 4.4. Assay of Replicative Lifespan of Hs68 Cells

The replicative lifespan of Hs68 cells was monitored by the cumulative-growth method [8]. Briefly, Hs68 cells were serially cultured in a 10 cm dish with 1.0 × 10^5^ cells per dish. The cells were subcultured exactly once per week, and the cell numbers were counted. The population doubling levels (PDLs) were calculated as log2 (Nt/No), where Nt and No were defined as the total count of cells at the time of harvesting and seeding, respectively. Cumulative PDLs (CPDs) were obtained by summing the total PDLs before a given passage number. The replicative lifespan was determined when the cells were senescent, which was defined as an Nt/No ratio less than 1.5 for two consecutive passages. The precise level of CPDs of Hs68 cells was not specified by the supplier; hence, we defined the CPDs at the initial passage as zero and used the additional CPDs to represent the doubling levels after the initial passage.

### 4.5. Determination of the Senescence-Associated β-Galactosidase (SA-βG) Activity

SA-βG activity was measured by the double-substrate assay [39]. Briefly, 1 × 10^4^ cells were cultured for 24 h in a 12-well culture plate. Then, the cells were fixed with PBS containing 2% formaldehyde and 0.2% glutaraldehyde (1 mL/well) for 5 min. After three washes, the fixed cells were incubated in a staining solution (1 mL/well) containing 2.45 mM X-Gal and 40 μM FDG in a humidified incubator at 37 °C for 24 h without CO_2_. The X-Gal-stained cells were imaged using a microscope. Alternatively, 100 μL of the supernatant was transferred to a 96-well plate for the determination of the fluorescein fluorescence by using a fluorimeter (Flx800, Bio-Tek, Winooski, VT, USA).

### 4.6. Handling Procedures for C. elegans

Nematodes were maintained and propagated on NGM as described elsewhere [40] with some modifications. In brief, nematodes were regularly grown at 23 °C in 10 cm plates in 20 mL/plate NGM; 10 mg (wet weight) of *Escherichia coli* OP50 (used as nematode food) in LB medium were spread onto the surface of the NGM plate and dried under laminar flow. The synchronization, preparation of NGM, LB medium, and M9 buffer (mainly for worm washing), and other procedures were performed as described previously [40].

Amp/FUdR plates with or without NA were prepared and subsequently used for the lifespan assay. Amp/FUdR plates were prepared using 5 cm plates by adding 10 mL/plate NGM containing 100 μg/mL ampicillin and 50 μM FUdR (to avoid subsequent egg hatching) [40]. In addition, 10 mg wet weight of OP50 bacteria in 200 μL of LB medium with or with NA was spread onto the center of the top surface of an NGM plate. After the surface bacteria buffer was dried, the Amp/FUdR plates were then expose to UV at two doses of 9999 × 100 mJ/cm^2^ to kill the bacteria on the surface of the plates.

### 4.7. Determination of the Intracellular NAD^+^ Level in C. elegans

Intracellular NAD^+^ in *C. elegans* was determined by the acid extraction method with subsequent enzymatic cycling [8]. Briefly, nematodes were collected and washed two times with M9 buffer in 15 mL centrifuge tubes (centrifuged at 1500 rpm for 5 min). Nematodes were extracted with a 4× volume of 7.0% ice-cold PCA with sonication. The extract (100 μL) was neutralized by an equal volume of 1 M K_2_HPO_4_. The NAD^+^ concentration in the extract was determined by a procedure similar to that described for the cycling assay in Hs68 cells. The intracellular NAD^+^ level in nematodes was corrected for the dilution factor at neutralization (i.e., twofold) and nematode volume (4× volume of the PCA solution). The intracellular NAD^+^ level in nematodes (μM) = the measured NAD^+^ (μM) × the dilution factor during neutralization (i.e., twofold) × 5, where 5 is obtained from (the PCA solution volume (μL) + the nematode volume (μL))/the nematode volume.

### 4.8. Assay of the Lifespan of C. elegans

The lifespan of the nematodes was determined as described elsewhere [40] with slight modifications. Synchronized larvae at the L1 stage were gently picked onto a 5 cm Amp/FUdR plate (30 worms/plate) containing various dosages of NA on the center of the top surface of the plate and grown at 23 °C. The plates of all groups were replaced with new plates every four days, and the surviving nematodes were counted every two days. The worms were considered dead when they did not react to the touch of a platinum wire or lost their pharyngeal pumping. The recorded survival percentage was plotted by Sigma Plot 8.0 and the average, median, and maximum lifespan values were calculated in SPSS.

### 4.9. Assay of the Pharyngeal Pumping in C. elegans

Pharyngeal pumping was determined as described previously [41,42]. On the third, fifth, and seventh days of the nematode lifespan, each group included 10 randomly selected nematodes to record pharyngeal pumping with a charge coupled device (CCD) video camera under a microscope. The number of pharyngeal beats within 30 s was calculated based on the slow-motion playback.

### 4.10. Determination of the Autofluorescence in C. elegans

Autofluorescence was determined as described previously [43,44]. On the 10th day of life, nematodes (*n* = 3) were randomly picked onto a slide coated with 2% agar pad (1 worm/slide). Nematodes were paralyzed with 20 mM sodium azide, and fluorescence of the nematodes was imaged by a ZEISS Axio Imager A2 fluorescence microscope (Zeiss, Göttingen, Germany) with a Rhod filter. Red autofluorescence was quantified by the ImageJ image analysis software (National Institutes of Health, Bethesda, MD, USA).

### 4.11. Assay of Body Bends of C. elegans

Body bends were determined by the swimming test as described previously [41]. On the 11th day of life, each group included 10 randomly selected nematodes to determine body bends. After crawling on an NGM plate without OP50 for 30 s to remove excess *E. coli* from the worms, each nematode was placed in a well of a 24-well plate containing 1 mL of M9 buffer. The motility of nematodes was recorded with a CCD video camera under a microscope, and the number of body bends within 30 s was calculated based on the slow-motion playback. Body bends were defined as the number of repeated twists at the center point of the nematode.

### 4.12. Determination of the SIRT1-Saturating Concentration of NAD^+^ In Vitro

The saturating concentration of NAD^+^ as a cosubstrate for SIRT1 was determined by using a commercial SIRT1 FRET-based screening assay kit (Cayman, Ann Arbor, MI, USA). All of the determination procedures, including the amount of the SIRT1 protein, were performed according to the manufacturer instructions, except the NAD^+^ concentration per assay was varied from 10 to 3000 μM.

### 4.13. Statistical Analysis

Data were analyzed using Student’s *t*-test or analysis of variance (ANOVA), followed by Duncan’s test for group mean comparisons using the SPSS v.14.0 software (SPSS, Inc., Chicago, IL, USA). The differences in the lifespan of the nematodes between the groups were analyzed using log-rank test. *p*-values less than 0.05 were considered statistically significant.

## 5. Conclusions

In summary, NA can extend the lifespan of *C. elegans* and improve aging markers, including pharyngeal pumping, autofluorescence, and body bends, when the steady-state level of NAD^+^ is approximately 55 μM. The lifespan extension and aging improvement by NA of *C. elegans* disappear when the intracellular level of NAD^+^ is increased up to 153 μM. Moreover, the steady-state level of intracellular NAD^+^ is approximately 460 μM, which is substantially higher than the SIRT1-saturating concentration of 200–250 μM; thus, NA cannot extend the lifespan of Hs68 cells. These results demonstrate that the lifespan extension ability of NA depends on whether the intracellular level of NAD^+^ is lower than the sirtuin-saturating concentration in Hs68 cells and in *C. elegans*. These results also suggest that the CRM potential of NA should be limited to individuals with lower intracellular NAD^+^, e.g., in individuals with vitamin B_3_ deficiency or the elderly.

## Supplementary Materials

Supplementary materials can be found at https://www.mdpi.com/1422-0067/21/1/142/s1.
