# Supplementary material for: The Lifespan Extension Ability of Nicotinic Acid Depends on Whether the Intracellular NAD+ Level Is Lower than the Sirtuin-Saturating Concentrations
Source: Int J Mol Sci. 2019 Dec 24;21(1):142. doi: 10.3390/ijms21010142 (PMC6982340; doi:10.3390/ijms21010142)
Supplement: Supplementary file 1 [file ijms-21-00142-s001.pdf]

Supplementary Materials:

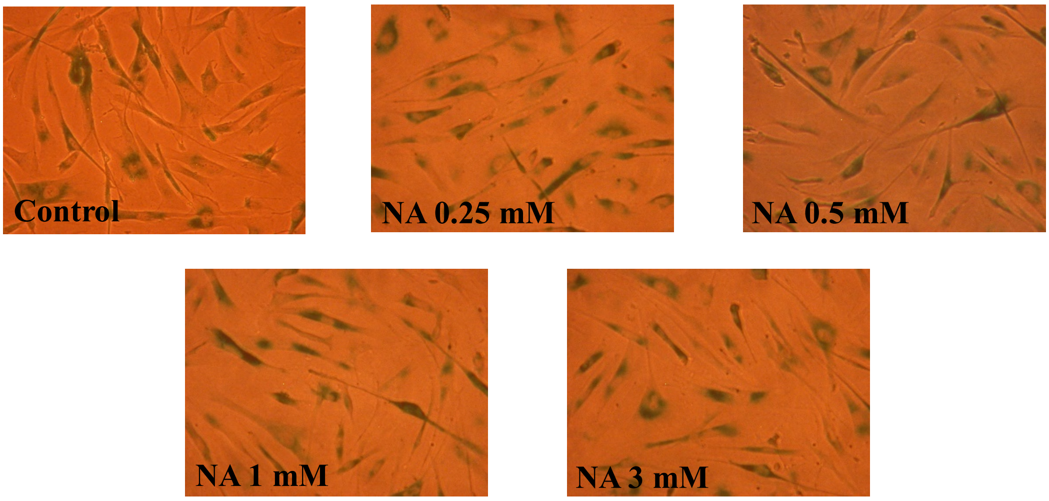

Figure S1. Effects of nicotinic acid (NA) on the senescence associated-β galactosidase (SA-βG) activity. Hs68 cells were serially cultured in the medium containing 0, 0.25, 0.5, 1 and 3 mM of NA. On day 91 of the serial culture, SA-βG activities were measured by the double-substrate method, and representative results from X-Gal staining were shown.

Table S1. Effect of nicotinic acid (NA) on the lifespan of *C. elegans.*

	**Mean lifespan (day)**	**Median lifespan (day)**	**Maximum lifespan (day)**	***P* value[#]**
Control	12.6 ± 4.3	12	22	
NA 100 nmol/plate	13.7 ± 4.3	14	22	0.192
NA 200 nmol/plate	13.6 ± 4.7	14	24	0.146
NA 600 nmol/plate	14.8 ± 4.7	16	24	0.003

[#]*P* values when compared to the control group by log-rank test.
Values for the mean lifespan were presented with mean ± SD, n = 90 for each group.

Table S2. Effect of nicotinic acid (NA) on the lifespan of *sir-2.1* mutants.

	**Mean lifespan (day)**	**Median lifespan (day)**	**Maximum lifespan (day)**	***P* value[#]**
Control *sir-2.1*	11.7 ± 4.0	10	20	
sir-2.1 + NA 600 nmol/plate	11.8 ± 4.7	12	20	0.698

[#]*P* value when compared to the control group by log-rank test.
Values for the mean lifespan were presented with mean ± SD, n = 90 for each group.

Table S3. Effect of nicotinic acid (NA) on the lifespan of *pme-1* mutants.

	Mean lifespan (day)	Median lifespan (day)	Maximum lifespan (day)	P value#
Control *pme-1*	13.8 ± 4.6	14	24	
pme-1 + NA 600 nmol/plate	15.2 ± 4.4	16	24	0.086

#P value when compared to the control group by log-rank test.
Values for the mean lifespan were presented with mean ± SD, n = 90 for each group.

Table S4. Effects of nicotinic acid (NA) on the lifespan of wild-type *C. elegans* grown on the NGM plates pretreated with NAD^+.

	Mean lifespan (day)	Median lifespan (day)	Maximum lifespan (day)	P_1#	P_2&
Control	12.2 ± 4.3	12	20		
Pre-add NAD^+ 100 μM	15.4 ± 4.9	16	24	<0.001	
Pre-add NAD^+ 100 μM + NA 600 nmol/plate	15.9 ± 4.9	16	26	<0.001	0.543

P_1 values when compared to the control group by log-rank test.
&P_2 value when compared between "Pre-add NAD^+ 100 μM" and "Pre-add NAD^+ 100 μM + NA 600 nmol/plate" groups by log-rank test.
Values for the mean lifespan were presented with mean ± SD, n = 90 for each group.
